# Extracellular Matrix from Porcine Small Intestinal Submucosa (SIS) as Immune Adjuvants

**DOI:** 10.1371/journal.pone.0027083

**Published:** 2011-11-07

**Authors:** Youssef Aachoui, Swapan K. Ghosh

**Affiliations:** Department of Biology, Indiana State University, Terre Haute, Indiana, United States of America; Instituto Butantan, Brazil

## Abstract

Porcine small intestinal submucosa (SIS) of Cook Biotech is licensed and widely used for tissue remodeling in humans. SIS was shown to be highly effective as an adjuvant in model studies with prostate and ovarian cancer vaccines. However, SIS adjuvanticity relative to alum, another important human-licensed adjuvant, has not yet been delineated in terms of activation of innate immunity via inflammasomes and boosting of antibody responses to soluble proteins and hapten-protein conjugates. We used ovalbumin, and a hapten-protein conjugate, phthalate-keyhole limpet hemocyanin. The evaluation of SIS was conducted in BALB/c and C57BL/6 mice using both intraperitoneal and subcutaneous routes. Inflammatory responses were studied by microarray profiling of chemokines and cytokines and by qPCR of inflammasomes-related genes. Results showed that SIS affected cytokine and chemokines microenvironments such as up-regulation of IL-4 and CD30-ligand and activation of chemotactic factors LIX and KC (neutrophil chemotactic factors), MCP-1 (monocytes chemotactic factors), MIP 1-α (macrophage chemotactic factor) and lymphotactin, as well as, growth factors like M-CSF. SIS also promoted gene expression of Nod-like receptors (NLR) and associated downstream effectors. However, in contrast to alum, SIS had no effects on pro-inflammatory cytokines (IL-6, IL-1β, TNF-α) or NLRP3, but it appeared to promote both Th1 and Th2 responses under different conditions. Lastly, it was as effective as alum in engendering a lasting and specific antibody response, primarily of IgG1 type.

## Introduction

Ninety years have passed since the concept of adjuvants took hold in vaccine design and is no longer regarded as “immunologist's dirty trick”. Thousands of chemicals have been assessed for their ability to enhance specific immune responses but the search is still on for broadly effective adjuvants. Ideally, a versatile and, preferably, biodegradable adjuvant should be safe and effective in engendering robust immune responses to a wide variety of pathogens and chemicals. It is not easy to produce an ideal, broadly effective adjuvant from a single compound. Vaccine efficacy does not depend merely on adjuvants but more importantly on the nature of the offenders that serve as immunogens. Adjuvants and immunogens together influence the host immune microenvironment, and thereby, modulate immunogenicity of a wide array of vaccines. However, no two adjuvants or immunogens interact in the same way, and the effects of adjuvants are subject to modifications by the immunogens or vaccines. In most cases, the precise mechanisms underlying the effects are unknown. Recently, there is a growing understanding that all known adjuvants function by affecting inflammation-responsive genes but they may differ significantly in their signature responses [1], [2]. These studies suggest that a better strategy to augment vaccine efficacy would be to incorporate a cocktail of adjuvants in the vaccine formulations rather than a single adjuvant chosen empirically. The mixture of adjuvants containing two or more compounds would complement or modulate individual effects with a broader and more beneficial impact on the host microenvironment and consequently on vaccine efficacy.

The making of adjuvant cocktails is not easy to achieve. One approach is to consider the mode of action of constituent cocktails, but that is not clearly understood. An alternative approach would be to use naturally occurring acellular structures, such as extracellular matrices (ECMs). ECMs are known to play diverse roles in cellular microenvironments. *In vivo*, they promote cell-to-cell interaction, angiogenesis, and immune extravasations [3], [4], [5]. As a biomaterial, they have found wide usage in wound healing and repair of urinary bladder defects, cardiovascular tissues, and ligament damage, etc. [6], [7], [8], [9], [10]. One such acellular ECM is SIS, a biomaterial from porcine small intestinal submucosa (Cook Biotech, IN, USA). It consists of predominantly collagens plus glycosaminoglycans, proteoglycans, fibronectin, b-FGF, and TGF-β, to name a few components [11], [12], [13]. Even though SIS is xenogenic in origin and, thus considered a xenograft in humans, it has been used for several years and evoked little ill effects, if any [14]. Its unique properties lie in its composition; the constituents are highly conserved proteins and may function as bioresponse modifiers or promote such responses. As a consequence, wound healing proceeds with tissue granulation and epithelization without the attendance of graft-versus-host reactivity [15], [16]. Most importantly, the particulate nature of SIS makes it readily amenable to phagocytosis by dendritic cells (DCs), which are the most efficient antigen-presenting cells (APCs), and hence, SIS is an attractive candidate for use as a cocktail of naturally occurring adjuvants.

Studies with SIS xenografts have revealed that when implanted, SIS elicits a vigorous immune response but the response is restricted to the Th2 pathway, which facilitates acceptance and remodeling of the graft material [17], [18]. Indeed, the Th2 dominance promotes efficient remodeling possibly by attenuating the pro-inflammatory cytokines induced by the Th1 pathway.

Recently SIS has been shown to enhance anti-prostate tumor immunity by evoking effective cell-mediated immunity [19]. Thus, it is increasingly evident that commercial SIS preparations could have a broader appeal as an adjuvant and for use in the making of conjugate vaccine preparations in a larger context. To determine whether this xenogenic products is equivalent to alum as an adjuvant, we asked the following questions: 1) does SIS impact the host's chemokine and cytokine microenvironment similar to alum, a prototypical adjuvant?; 2) does SIS involve the inflammasome-related core-adjuvant genes, as does alum, in recruiting innate immunity?; and 3) does SIS, like alum, enhance antibody responses to soluble, non-self-protein vaccines and hapten-protein conjugate (the latter as a prototype of conjugate vaccines)?.

In this study, the evaluation of SIS adjuvanticity was carried out in BALB/c and C57BL/6 mice via intraperitoneal and subcutaneous routes in the presence and absence of Ovalbumin (OVA) and phthalate-Keyhole limpet hemocyanin (KLH) immunogens. Inflammatory responses were studied by microarray profiling of cytokines and chemokines and by qPCR of inflammasomes-related genes. Results demonstrated that SIS up-regulated IL-4 and CD30-ligand and promoted the gene expressions of Nod-like receptors (NLR) and associated downstream effectors, implying modulation of cytokine and chemokine expression through these receptors. SIS moderately up-regulated expressions of chemotactic factors LIX, KC (neutrophil chemotactic factors), MCP- 1 (monocyte chemotactic factor), MIP 1-α (macrophage chemotactic factor), fractalkine, lymphotactin, and growth factors like M-CSF. Overall, SIS seemed intrinsically strong and was as effective as alum in engendering a Th2 type mediated antibody response, primarily of IgG1 type. However, SIS differed from alum by provoking no pro-inflammatory cytokines (IL-6, IL-1β, TNF-α) or NLRP3.

## Materials and Methods

### Vaccine formulation

Vaccine formulation consisted of either ovalbumin (OVA) (100 µg/mice) or phthalate- KLH conjugate (100 µg/mice) prepared as described by Ghosh et *al* [20]. The antigens were emulsified with two commercial SIS preparations (SIS-H and SIS-M, so named to identify two lots of the commercial preparation) provided by Cook Biotech as follows: 200 µL of antigen (100 µg) plus 5 mg (SIS-H or SIS-M) in 250 µL PBS containing an emulsifier, Arlacel A (15%) (Acros organics, Thermo Fisher scientific, NJ, USA). SIS particulate was produced from hydrated sheets of clean, disinfected, decellularized SIS. The sheets were cut into small pieces, and kept frozen at or below −80°C. After grinding through a cryogenic hammer mill into a range of particles, they were freeze-dried and sieved for sizing. The particles, we used, were in the 45–150 micron size range. Details of SIS particle preparation appeared in several publications and patents [21], [22]. For comparison, commercial adjuvants such as alum and CFA/IFA or no adjuvant were used. Inoculums containing commercial Adjuvants were prepared as follows: 200 µL of antigen (100 µg) was mixed to 200 µL incomplete of Freund's adjuvants (CFA) or (IFA) (Sigma Chemical Co., St. Louis, MO, USA) or adsorbed on alum (76 µL alum+ 124 µL PBS) (Sigma Chemical Co) by mixing vigorously by vortexing a few times using a syringe.

### Mice

Female BALB/c, C57 BL/6 mice, 6–8 weeks of age, were used throughout the study. The study was reviewed and approved by the Institutional Animal Care and Use Committee (IACUC) of Indiana State University under the protocol (ID#09-15-2010:SKG/YA). Pathogen-free BALB/c mice were bought from Harlan Sprague-Dawley (Indiana, USA), kept in quarantine for two weeks before relocation to specific mouse facility that is monitored by veterinarians, IACUC and USDA inspectors. Pathogen-free C57BL/6 mice were purchased from the Jackson laboratory (Maine, USA), and used after their release from quarantine. Information on microbial pathogens can be found in the vendors' websites (www.harlan.com & www.jax.org).

### Immunization

Mice (n = at least 6 in each experiment) were grouped as: (1) PBS group receiving only antigen but no adjuvant; (2) CFA/IFA (antigen plus adjuvant; first CFA, then IFA in subsequent immunizations); (3) Alum; (4) SIS-H; and (5) SIS-M. Two types of antigens were tested: ovalbumin (OVA) and phthalate-KLH, a hapten- protein conjugate. Adjuvant administrations, with or without immunogens were performed intra-peritoneally (i.p.) or subcutaneously (s.c.). Mice were given two booster immunizations at 10-day intervals and four months later, mice were given a booster immunization with antigen alone. Five days after each immunization mice were bled under anesthesia through the retro-orbital plexus. Induced serum antibodies were assayed by ELISA. For determination of cytokines and chemokines or their real time gene expression RT-PCR, mice (at least n = 3) were given the adjuvants i.p in 500 µL PBS and peritoneal lavages were collected 24 hours later.

### ELISA analysis

Determination of anti-phthalate, anti-KLH, or anti-OVA antibodies was done by enzyme linked immunosorbant assays (ELISA). Flat-bottomed Corning plates were coated for 2 h at 37°C with 50 µL of 10 µg/mL of either phthalate-BSA conjugate, KLH or OVA. The plates were washed four times with PBS containing 0.01% Triton X-100, blocked overnight with 1% BSA and washed again. Various dilutions (10^1^–10^5^) of test sera from normal and immunized mice were added in triplicate to the plates and incubated at 37°C for 1 hr. Following incubation and after washing four times with PBS/Triton X-100, rabbit anti- mouse immunoglobulin-horse-radish peroxidase (HRP) (50 µL) (1∶ 3000 dilution) was added. Plates were incubated for 1 hr, washed again and developed using o-phenyl diamine (OPD). The reaction was stopped by adding 50 µL of 10% H2SO4 and the intensity of color was determined at OD 490 nm using a Microplate Elisa Reader (Bio-Tek instruments, VT, USA).

To determine anti-DNA antibodies, ELISA plates were pre-coated for 2 hrs at 37°C with 50 µl of methylated-BSA (50 µg/mL). The plates were washed four times, coated with calf thymus DNA (10 µg/mL), incubated for 2 hrs at 37°C, and assessed as described above.

To determine isotypes of antibodies produced (IgM, IgG1, IgG2a, IgG2b, and IgG3), individual mouse sera from various groups of immunized mice were diluted to 1/1000 and tested in triplicate according to the manufacturer's protocol (Southern Biotech, Alabama). Plates were coated with either phthalate-BSA, or OVA as described above

### Analysis of cytokines and chemokines

BALB/c mice were injected with adjuvants and peritoneal exudates were collected after injecting with 3 ml of PBS; peritoneal exudate cells (PECs) were spun down and used for gene expression profiling by RT-PCR. The supernatant fluid (1 ml) was utilized for determination of cytokines/chemokines using mouse inflammatory cytokine array kits and protocols from Ray Biotech Inc., GA, USA. Signal intensities were quantified and analyzed from the array images on the membrane using Image J software from NCBI [23]. Positive and negative controls from six array spots were used to normalize the results from different membranes. For each spot, the net optical density level was determined by subtraction of the background density from that of the sample spots, divided by the values of positive control density. Levels of cytokine were expressed as percent intensities (RI) relative to the positive control provided in the membranes.

### Analysis of gene expressions by quantitative real-time qRT-PCR array

Isolated PECs were used for profiling gene expression by real-time qRT-PCR. Briefly, total RNA was isolated from all samples according to the manufacturer's recommendations (Ambion, TX, USA). The RNA preparation was considered to be good quality when it's OD 260/280 and 260/230 ratios approached 2. Equal amounts of RNA (1 µg), from all sample groups, were reverse-transcribed using RT2 first-strand kit from SA Biosciences (Frederick, MD). The cDNAs were then labeled by RT2 Real-Time SYBR Green PCR Master Mix (SA Biosciences, MD, USA)) as indicated in RT2 Profiler PCR Array protocol (SA Biosciences). A 25 µL aliquot of this mix was loaded onto the wells of PCR array plates (PAMM-97, SA Biosciences) and PCR was performed on a Stratagene Mx3000P cycler using a cycling program provided by the manufacturer. Relative changes in gene expressions were calculated and analyzed using SA Biosciences' (Maryland, USA) web-based PCR array data analysis methodology (http://pcrdataanalysis.sabiosciences.com/pcr/arrayanalysis.php website). Gene expressions were normalized with respect to five house-keeping genes included in the array kit and calculated as averages of log_2_ ratios. The array evaluated expressions of 84 genes involved in inflammasome pathway. Genes of mice that differed by 1.5-fold compared to buffer controls were considered as adjuvant-mediated inflammation-responsive genes.

### Statistical analysis

One-way ANOVA and Student's t-test (SPSS software) were used to determine statistical significance. Levels of p<0.05 were considered statistically significant. Data are expressed as mean ± SD.

## Results

### Chemo-attractants and cytokines in BALB/c in response to SIS adjuvants

Wound healing and tissue remodeling are facilitated by SIS biomaterials possibly by virtue of their ability to initiate and sustain favorable cytokine milieu. In a previous report, we showed that intraperitoneal immunization with alum, an adjuvant licensed for use in humans, promotes a pro-inflammatory microenvironment characterized by induction of cytokines (IL-4, IL-12p40p70, IL-1α, sTNF R I, sTNF R II) and chemotactic factors (MCP-1, LIX, KC, M-CSF, and TIMP-1) [24]. To assess whether SIS provokes inflammatory reactions, we studied SIS alone in peritoneal exudates 24 hours after i.p. administration. Two preparations of porcine small intestinal submucosa, SIS-H and SIS-M were evaluated.

**Figure 1:**
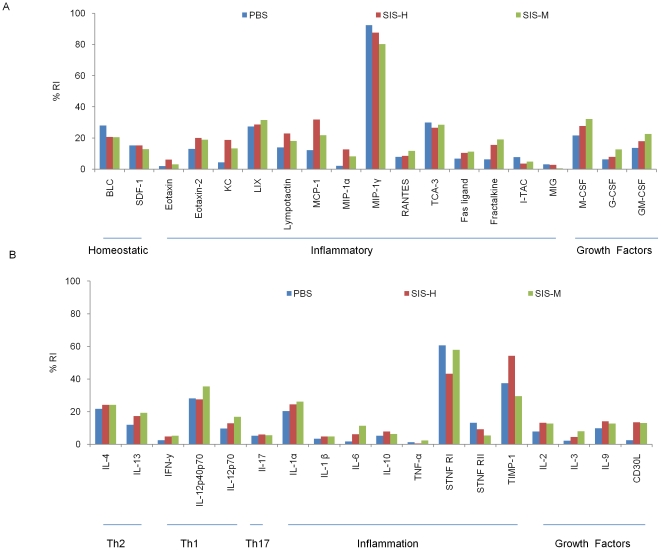
Effects of porcine small intestinal submucosa (SIS) on chemokine and cytokine protein microenvironment in BALB/c peritoneal exudates. BALB/c mice were injected i.p. with SIS preparations (SIS-H or SIS-M) without the antigens and peritoneal exudates harvested after 24 hr. Control groups were treated with PBS buffer. Peritoneal fluids were assayed to determine chemokine and cytokine expression as detailed in Materials and Methods. Data are expressed as the mean relative intensity relative to positive control of each chemokine or cytokine protein detected using pooled peritoneal fluids of 3 mice per group in duplicate. The result is an average of two separate experiments.

The results in Fig. 1 show that both SIS-H and SIS-M were similar to alum in inducing a plethora of cytokines and chemokines including leukocyte chemotactic factors (KC, LIX, eotaxin-2, MCP-1, lymphotactin, fractalkine, MIP-γ), macrophage chemotactic factor (MIP-1α), eosinophils chemotactic factor (eotaxin-2), fractalkine (a potent chemo attractant for T cells and monocytes) and FASL (involved in apoptosis). Clearly, this implied immune mobilization by recruitment and activation of neutrophils, eosinophils, monocytes, and macrophages. In all experimental groups, there was also a modest increase in the levels of growth factors GM-CSF, M-CSF, G-CSF, which help in the differentiation of monocytes to DCs, macrophages, and granulocytes [25], [26], [27]. Induction of moderate levels of IL-1α, and sTNF R I from SIS preparations suggested that there may be some tissue injury. However, such injury seemed minor due to the fact that there was no attendant increase in pro- inflammatory cytokines such as IL-6, IL-1β, IL-10, TNF-α and IL-17. The influence of SIS preparations was noticed on cytokines associated with the induction of both Th1 and Th2 cells. The SIS preparations evoked Th2 cytokine (IL-13, IL-4), as well as Th1 polarizing cytokine (IL-12 P70P40 and IL-12 P40). SIS also influenced cytokines IL-2, IL-9 and CD40L necessary for stimulation and growth of T helper cells.

### Effects of adjuvants on inflammatory gene expression

**Figure 2:**
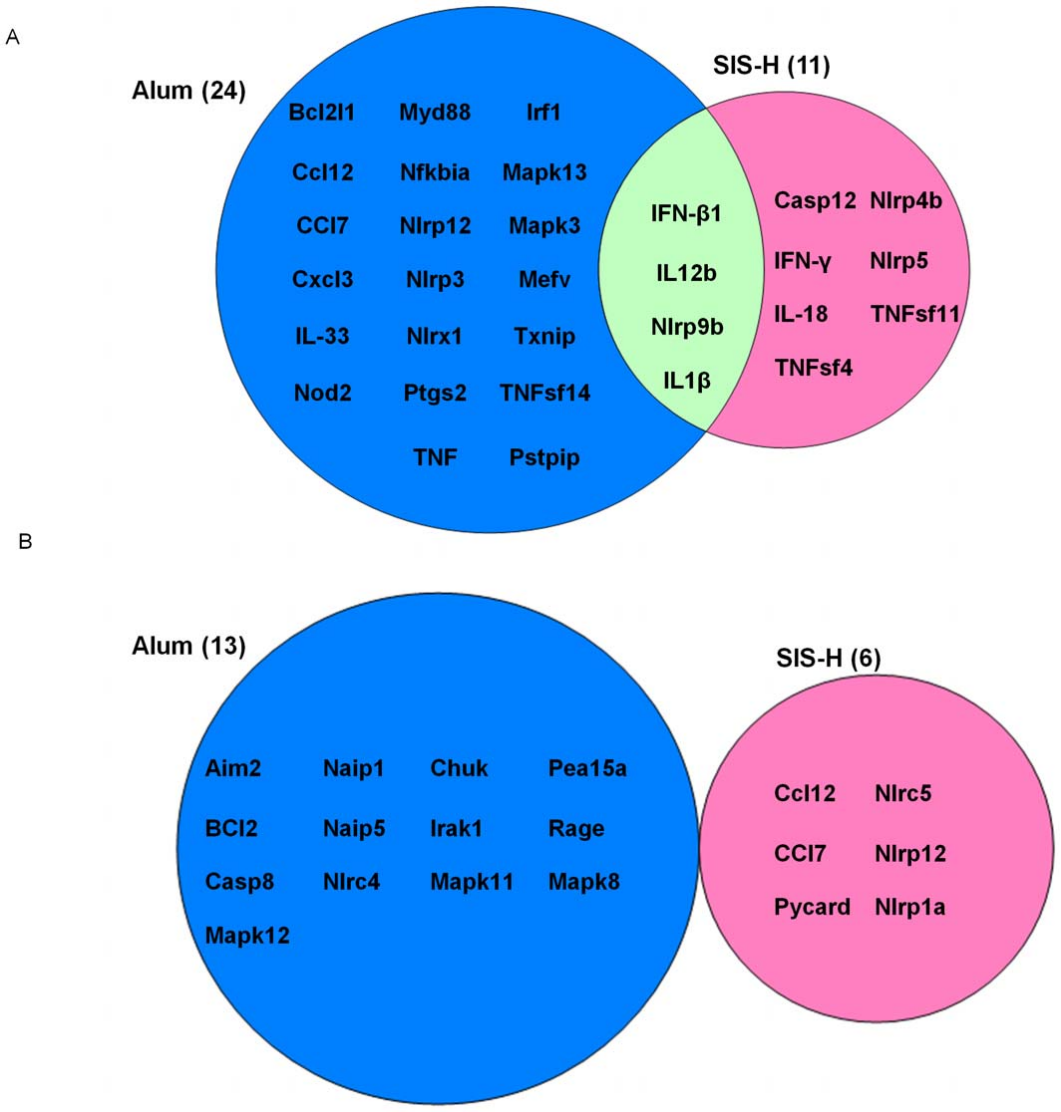
RT-PCR Microarray analysis of transcriptome profiles of inflammatory genes induced by vaccine adjuvants alone in mouse peritoneum. Eighty-four genes were assessed and those genes up-regulated (A), or down regulated (B) with an average log2 ratio ≥1.5 were selected and plotted as a Venn diagram. Results for the alum-treated group refer to our previous study [24].

The innate immunity is known to be strategically involved in initiating inflammatory processes as a response to “stranger or danger signals” from adjuvants and antigens [28], [29]. We hypothesized that SIS like alum might function by interacting with the receptors of the innate immunity system, particularly those implicated in inflammatory processes such as Nod-like receptors (NLRs). In recent reports, NLR-associated gene activation pathways have been shown to play crucial roles in the adjuvanticity of alum and MF59 [30], [31]. To address whether adjuvanticity of SIS would follow the same pathway, we focused on gene expression profiling of NLRs in mice. Using RT-PCR microarray, we measured the expression of 84 genes in BALB/c mice immunized i.p. with SIS-H or alum alone. Our results shown in Table 1 and 2 indicate that SIS alone significantly affected 17 inflammation-responsive genes. Among these genes, there were 11 genes up-regulated above 1.5 fold on a log_2_ scale (table 1) and 6 genes down-regulated below 1.5 fold (table 2). Genes responding positively to SIS included Nod-like receptors NLRP4b, NLRP 5 and NLRP9b, and cytokine genes associated with Th-1 response such as INF- γ, IL-12b, INF-β1 and IL-18. Furthermore, SIS up-regulated expressions of inflammatory genes IL-1β, caspase 12, TNFsf 4 and TNFsf 11. Interestingly, SIS had no effect on NLRP3, Txnip and Pstpip that were shown previously to participate in alum-mediated NLRP-3 inflammasomes [32], [33]. In a previous study, we showed that alum could up-regulate 24 inflammation- responsive genes that include NLRP3 and Txnip [24] that were shown previously to form or participate in formation of inflammasome platforms [32], [33]. Moreover, unlike SIS, alum activated chemotactic factor CCL12, CCL7, and CXCL3, inflammatory cytokine TNF, and downstream effectors genes such, MyD88, Mefv, and Ptgs2. Alum also induced Mapk3 and MapK13, two downstream signaling factors for MAPK pathway and interferon-regulatory factor (Irf1); the latter two of which may be involved in the production and regulation of pro-inflammatory cytokines [24]. Comparison of genes activated by SIS-H and alum is summarized as a Venn diagram (Fig. 2).

**Table 1:** RT-PCR Microarray analysis of transcriptome profiles of inflammatory genes induced by vaccine adjuvants alone SIS-H in mouse peritoneum.

Gene	Fold regulation	Gene	Fold regulation
IFN-β1	2.4	Nlrp6	1.2
IL-12b	2.3	Mefv	1.2
Nlrp9b	2	Ciita	1.2
Nlrp5	1.8	Irf1	1.2
Casp12	1.7	Traf6	1.2
TNFsf4	1.7	Ctsb	1.1
IL-1β	1.7	Txnip	1.1
IFN-γ	1.6	Xiap	1.1
Nlrp4b	1.5	Ikbkb	1.1
IL-18	1.5	Ikbkg	1.1
TNFsf11	1.5	Irak1	1.1
IL-12a	1.4	Chuk	1
IL-33	1.4	Irf2	1
Cd40lg	1.4	Mapk8	1
Tnf	1.3	Cxcl3	1
Nlrp4e	1.3	Birc3	1
Ptgs2	1.3		

Eighty-four genes were assessed and genes up-regulated are presented.

**Table 2:** RT-PCR Microarray analysis of transcriptome profiles of inflammatory genes induced by vaccine adjuvants alone SIS-H in mouse peritoneum.

Gene	Fold regulation	Gene	Fold regulation
Ccl12	−1.7	Nfkbia	−1.2
Nlrc5	−1.7	Nfkbib	−1.2
Nlrp12	−1.5	Mapk13	−1.2
Nlrp1a	−1.5	Panx1	−1.1
Pycard	−1.5	Pea15a	−1.1
Ccl7	−1.5	Pstpip1	−1.1
Nod2	−1.4	Rage	−1.1
Bcl2l1	−1.4	IL-6	−1.1
Naip1	−1.4	Ripk2	−1.1
Cxcl1	−1.3	Tirap	−1.1
Nlrx1	−1.3	Birc2	−1.1
Map3k7ip1	−1.3	Myd88	−1.1
Irf3	−1.3	Mapk12	−1.1
Bcl2	−1.3	Map3k7ip2	−1.1
Naip5	−1.3	Mapk3	−1
Nlrp3	−1.3	Mapk11	−1
P2rx7	−1.2	Ccl5	−1
Casp8	−1.2	Mapk9	−1
Card6	−1.2	Map3k7	−1
Casp1	−1.2	Rela	−1
Aim2	−1.2	Fadd	−1
Mapk1	−1.2	TNFsf14	−1
Nlrc4	−1.2	Cflar	−1
Nfkb1	−1.2		

Eighty-four genes were assessed and genes down-regulated are presented.

### SIS adjuvants promote high levels of anti-OVA response mediated by T helper type-2 cells

**Figure 3:**
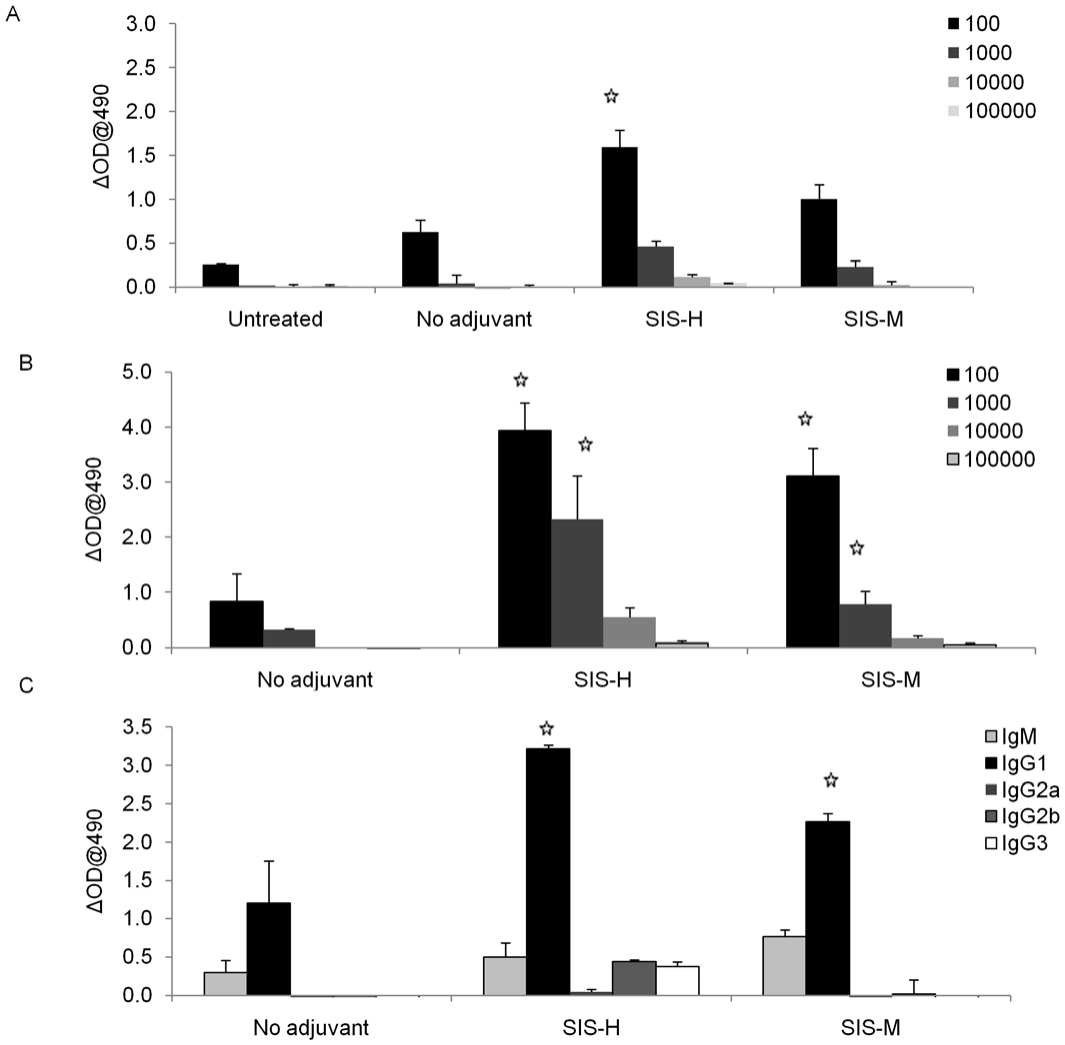
Effects of SIS adjuvants on anti-ovalbumin antibody response in C57BL/6 mice. Mouse serum samples were collected on day 5 after (A) 2nd, and (B) 3rd immunizations with OVA plus adjuvants as described under Materials and Methods. Antibody responses were assessed using ELISA, (C) determination of IgG sub-classes of anti-OVA antibodies, induced with OVA containing adjuvants, in sera from the 3rd immunization. Commercial isotyping kits (Southern-Biotech) were used to perform ELISA in serum samples (starting at dilution 1∶1000). The results represent mean ± SD (n = 6 mice per group in two separate experiments). The significance in experimental groups relative to the group given antigen only (no adjuvant group) was calculated at the level of p≤0.05.

The above study showed that both SIS and alum influence the host microenvironment by modifying expression of innate immunity-related chemokine and cytokine genes, although there were characteristic differences between the two adjuvants. These observations led us to question whether these effects on innate immunity would help activate acquired immunity and augment vaccine efficacy. Using OVA as the model antigen in our vaccine formulations, SIS was assessed for its effectiveness in anti-OVA antibody induction in C57BL/6 mice. The results in Fig. 3 revealed significant booster effects; clearly SIS H or SIS M were highly effective at increasing the magnitude and titer of OVA-specific antibody, particularly high levels of IgG1 antibody subclass. This suggests that SIS biomaterials effectively promoted a Th-2 response.

### SIS adjuvants promote antigen-specific antibody response but no measurable autoimmune effects

**Figure 4:**
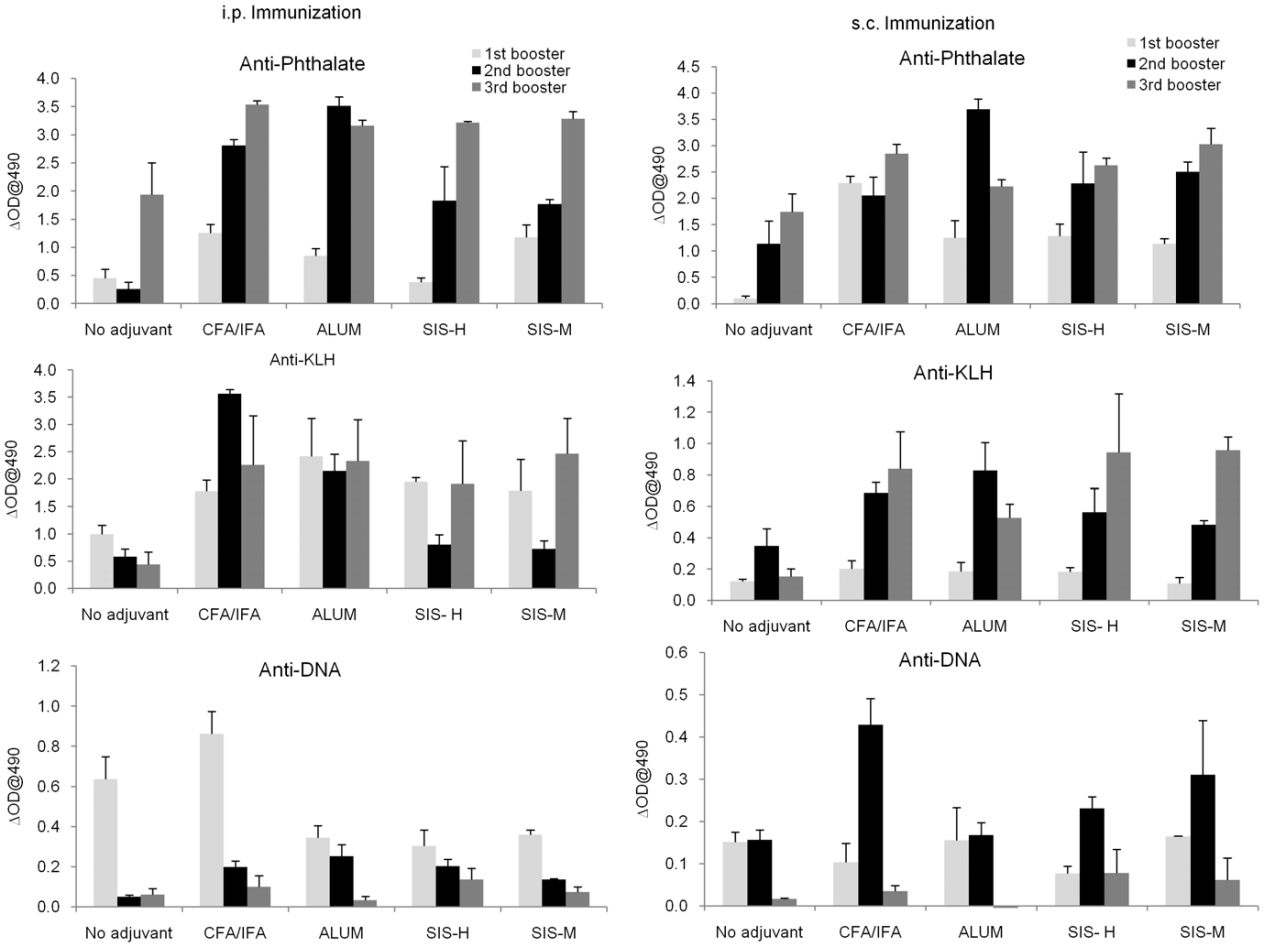
Evaluation of phthalate-KLH antibody response in BALB/c mice. Mice were immunized with phthalate-KLH conjugate emulsified in different adjuvants. Serum samples were collected as described under Materials and Methods and diluted to 1∶1000 in 0.5% PBS/BSA. Anti-phthalate, anti-KLH, and anti-DNA antibody levels were determined using ELISA as described. The results represent the mean ± SD (n = 6 mice per group in two separate experiments). The significance in experimental groups was determined relative to the group given antigen only (no adjuvant group) at the level of p≤0.05.

Next, we addressed whether the adjuvanticity of SIS biomaterials would vary depending on mouse strains, routes of immunization (i.p. or s.c.) or antigenic differences. In addition, we tested whether immunization with SIS would cause or aggravate autoimmune responses, a side effect shown to be associated with the use of many adjuvants [34]. This study was performed in non-immune prone BALB/C using phthalate-KLH conjugate as the experimental antigen administered by intra peritoneal or subcutaneous routes. Previously, we showed that phthalate, a plasticizer and a common environmental hazard, would cause a lupus-like syndrome in NZB/WF1 mice and the response was greatly aggravated by some adjuvants [35], [36]. The response to phthalate-KLH is characterized by an antibody response that is mostly directed toward the hapten, the phthalate moiety, in the conjugate with concomitant induction of cross-reactive anti-DNA antibodies. In BALB/c mice also, there was initially some increase in anti-DNA antibody but was down-regulated during subsequent booster immunizations. Our results, as shown in Fig. 4 indicate that, irrespective of the routes of immunization, BALB/c mice immunized with phthalate-KLH as emulsions with SIS-H or SIS-M developed high titer antibody responses against phthalate-KLH. Although the magnitude of this response was slightly less than that induced by alum or CFA/IFA, it was directed against both phthalate and KLH; the phthalate cross-reactive anti-DNA antibody levels were insignificant in SIS-immunized mice.

**Figure 5:**
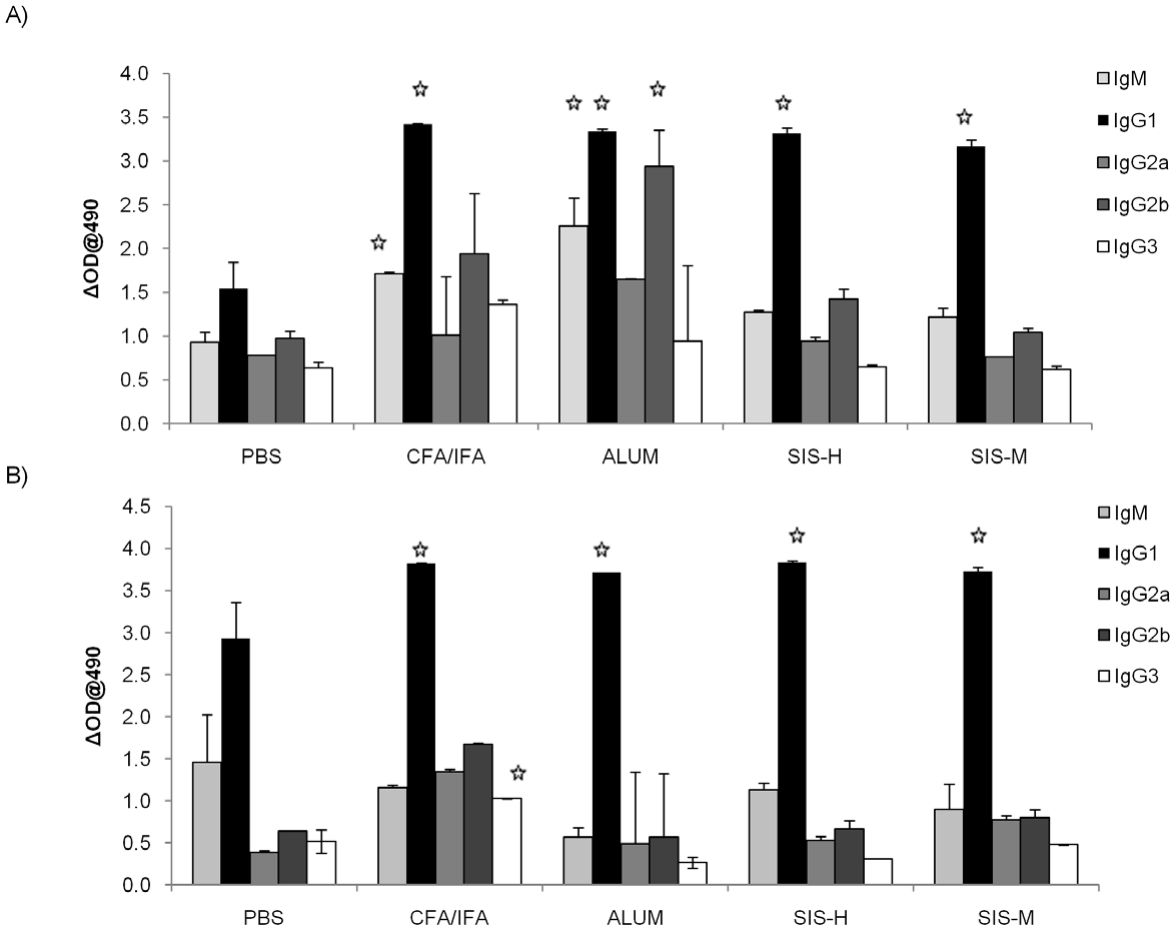
Determination of IgG sub-classes of anti-phthalate antibodies induced with phthalate-KLH conjugates in different adjuvants. This was done using commercial ELISA isotyping kits with serum samples (at dilution 1∶1000) collected after (A) intraperitoneal, or (B) subcutaneous immunization as described in Materials and Methods. Results represent mean ± SD (n = 6 mice per group in two separate experiments). The significance in experimental groups was determined relative to the group given antigen only (no adjuvant group) at the level of p≤0.05.

Analysis of IgG isotypes directed against the phthalate moiety (Fig. 5) also showed that SIS adjuvants like alum and CFA/IFA promoted IgG1 by both i.p. and s.c. routes. However, unlike alum and Freunds' adjuvants, SIS was less efficacious in inducing IgG2a and IgG2b responses particularly after i.p. injection.

## Discussion

This study evaluated adjuvanticity of an acellular biomaterial derived from porcine small intestinal submucosa (SIS), a commercial product licensed primarily for use in surgical procedures dealing with wound healing in humans [7], [8], [9], [10]. Despite its xenogenic nature, SIS works well in humans in vivo and causes no detrimental inflammatory effects. Indeed, it greatly facilitates the process of wound healing. Though largely a collagenous product, SIS is known to contain minute amounts of other proteins typically associated with such extracellular matrices [11], [12], [13]. This unique and highly sterile biomaterial has been of interest for use as a type of multi-protein naturally-occurring biodegradable cocktail adjuvant. Indeed, its adjuvanticity has already proved effective in the successful development of a model prostate cancer vaccine [19].

Immune enhancement by adjuvants, however, can work both ways: it could be ameliorating or detrimental. Therefore for a biomaterial or a compound to be an adjuvant, safety is certainly the primary concern. One way to assess safety is to determine whether the test material causes physical or behavioral problems such as splenomegaly or discomfort. This issue is irrelevant for SIS as it is widely used as a safe biomaterial for wound healing in animals and humans. In the context of adjuvanticity, we addressed the safety issue in terms of important parameters associated with inflammatory or immunodulatory cytokine/chemokines microenvironment and consequent activation of acquired immunity. We tested the safety and efficacy of two batches of SIS biomaterials from Cook Biotech, SIS-H and SIS-M alone and in combination with soluble protein antigens in inbred strains of mice, C57BL/6 and BALB/c. We evaluated micro- environmental changes (cytokines and chemokines) brought about by SIS relative to alum and the resulting impact on the quality and titer of specific antibody response.

Our study of the chemokine/cytokine milieu both at the protein and gene expression levels revealed both similarities and differences in the effects of SIS and alum. It is apparent that SIS preparations do have inflammatory effects, albeit to a lesser degree than alum, possibly because SIS by virtue of its composition is biodegradable and thus less consequential. Interestingly, alum activates more genes than SIS, and these genes comprise those of several nod-like receptors (NLRs) including NLRP3 inflammasome, a molecular platform that is required for caspase- dependent cleavage of cytokines such as IL-1β, IL-18 and IL-33 [37], [38]. Similar to alum, SIS up-regulates IL-1β but it does not affect NLRP3. In addition, alum also activates downstream effectors, Txnip, Irf1, Mapk3 and 13 indicating a strong inflammatory response. In contrast, SIS up-regulates a lesser number of NLRP genes such as NLRP4b, NLRP5 and NLRP9. It also up- regulates TNFsf4, TNFsf11, INF-β1 and INF-γ cytokine genes; these are involved in proliferation and activation\of innate and adaptive immunity. Interestingly, SIS does not evoke pro-inflammatory molecules such as IL-6, TNF-α and IL-17. Both SIS and alum have similar impacts on chemokines, such as LIX and KC (neutrophil chemotactic factors), MCP-1 (monocyte chemotactic factors), BLC (B lymphocyte chemotactic factor), and MIP 1-α (macrophage chemotactic factor). In addition, SIS adjuvants, with the same intensity as alum promote the expressions of growth factors GM-CSF, G-CSF, M-CSF. These growth factors are important for differentiation and maturation of monocytes and granulocytes that develop to become mature antigen-presenting cells [25], [26], [27]. Alum reportedly has the ability to activate monocyte- derived APCs, which in turn would promote isotype-switching and high-titer antibody response [39], [40]. Alum also helps recruitment of granulocytes which play an auxiliary role in enhancing this response [41]. From our results, SIS adjuvant is similar to alum in provoking monocytes and granulocytes, and this may explain how it enhances antibody response. Furthermore, recruitment of APCs following SIS administration is accompanied by production of cytokines such as IL-4 and CD 40 L, which are crucial for priming of CD4+ T helper cell and consequently for isotype switching that occurs during B cell response [42], [43].

When the SIS biomaterial was used with antigens ovalbumin and phthalate-KLH, there was significant enhancement of anti-OVA and anti-phthalate responses and seemed to be mediated by a Th2 response. These results were in agreement with previous reports implying that SIS when used as the biomaterial for wound healing purposes would bolster the Th2 environment and produce mild inflammatory response [17]. Interestingly, when tested alone, SIS adjuvant up-regulates the gene expression of IL-18, IL-12b, and INF-γ, cytokines that induce Th-1 cells. This suggests a dichotomy in the action of SIS: a Th2 response in the presence of non-self immunogens like OVA or hapten-protein conjugate, and a Th-type1 response in their absence. This may explain a previous report by Suckow, et *al*. that showed that prostate tissue vaccine stimulated a Th1 response and specifically prevented prostate cancer growth [19], [44]. In a future study, it will be of interest to assess the effects of SIS, with or without non-self antigens, on expressions of co-stimulatory molecules by antigen-presenting cells.

In conclusion, SIS adjuvant augments immunogenic potential of soluble proteins without inducing any pathologic inflammatory response. It is safe and effective. Most commercial adjuvants are known to function by inflicting inflammatory tissue damage at sites of injection. This may have unintended consequences such as autoimmune disorders as noted in the use of established adjuvants like complete Freunds adjuvant, which cause inflammation and tissue necrosis with formation of granuloma in lung and kidney [45]. Squalene in MF59 and alum also induce inflammatory response; squalene is linked to autoimmune responses in rodents [34] and alum to dementia [39]. In contrast, SIS has been used as a biomaterial for several years and has a clean record. It is also safe and effective as an adjuvant even with soluble protein antigens. In our ongoing study, we are assessing whether SIS would be useful in vaccination of autoimmune prone mice such as NZB/WF1 mice without aggravating lupus-like syndromes. Furthermore, it would be of interest to determine whether the effectiveness of SIS adjuvant can be further improved by co-administration with alum or other approved adjuvants like squalene or bioresponse modifiers.
